# Chronic Type B Aortic Dissection With Aneurysmal Dilatation Presenting With Recurrent Seizures: A Case Report

**DOI:** 10.7759/cureus.91915

**Published:** 2025-09-09

**Authors:** Parto Siavosh, Jaafar Al-Sadiq Jaf, Seyed Hossien Shaker

**Affiliations:** 1 Hematology and Medical Oncology, Rasoul Akram Hospital, Tehran, IRN; 2 Stroke, Stepping Hill Hospital, Manchester, GBR; 3 Emergency Medicine, Iran University of Medical Sciences, Tehran, IRN

**Keywords:** aortic aneurysm, aortic dissection, neurological symptoms, neurology and critical care, seizure

## Abstract

Aortic aneurysms can remain asymptomatic or present with complications, including aortic dissection. Aortic dissection itself may manifest with a wide spectrum of signs and symptoms, which can make diagnosis challenging.

A 63-year-old man is discussed in this article, who presented with episodes of seizure. He had a history of type B aortic dissection being treated medically. Chest computed tomography (CT) scan showed a 10 cm aortic aneurysm and dissection. He was admitted to the coronary care unit and underwent thoracic endovascular aortic repair (TEVAR). His postoperative course was uneventful, and he was discharged home in stable condition.

One of the most important but easy-to-miss diagnoses to consider in patients with neurological symptoms is aortic dissection. Depending on the type and severity, and chronicity of the disease, different management plans can be considered. TEVAR is one of the new techniques in treating this condition.

## Introduction

The aorta is the main and largest vessel in the body. Aortic dissection happens when the weakened wall of the aorta tears, causing blood to leak between the layers that make up the walls of the arteries. This can happen suddenly or slowly over time [1]. Aortic dissection can present with various signs and symptoms, including a sudden, severe pain across the chest, often felt in the back or between the shoulder blades; pain in the jaw, face, abdomen, back, or lower extremities; feeling cold, clammy, and sweaty; fainting; and shortness of breath [1]. It is very important for clinicians to understand these different presentations of aortic dissection. This case report highlights chronic presentations and emphasizes the importance of considering aortic dissection among the list of vital diagnoses.

## Case presentation

A 63-year-old man presented to the emergency department (ED) on April 25, 2024, complaining of an episode of generalized tonic-clonic (GTC) seizure (characterized by loss of consciousness with stiffening and rhythmic jerking of the limbs). He had an episode of tonic-clonic seizure at 6 AM and came to the emergency room (ER) at 2 PM fully conscious, with no symptoms on admission. He also complained of a headache in the vertex area starting 2-3 days prior to admission. His headaches usually lasted 20 minutes and had no radiation, and also did not respond to nonsteroidal anti-inflammatory drugs (NSAIDs).

The patient had a previous history of serial seizures starting eight years ago after an occipital lobe hemorrhage (of unknown cause), followed by an ischemic cerebrovascular accident (CVA) dating back five years ago. He also had a history of aortic dissection type B, found accidentally during one of his admissions for a seizure. As the dissection was type B, he was a candidate for oral agent treatment and was discharged home symptom-free.

**Figure 1:**
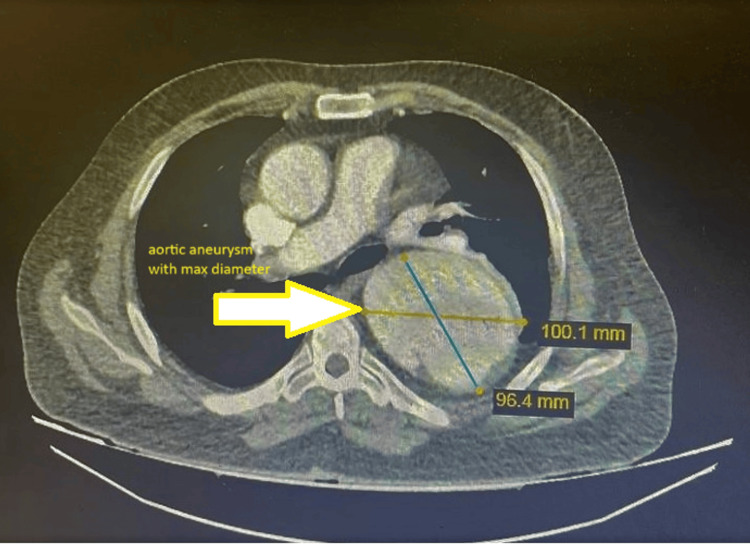
First chest CT scan (pre-operation) This CT shows the thorax, demonstrating a huge thoracic aortic aneurysm (about 100 mm diameter). Such a large dilatation is at very high risk of tear and mandates urgent intervention, typically with TEVAR. CT: computed tomography, TEVAR: thoracic endovascular aortic repair

First, an electrocardiogram (ECG) was taken, which showed a normal sinus rhythm and no abnormalities. During the first three hours of admission, the patient had one episode of GTC seizure in the ED. He underwent a spiral brain and chest CT scan without contrast. The brain CT showed no signs of acute hemorrhage or ischemia. The chest CT scan showed an aortic aneurysm, which has also been dissected (from the descending aorta), with its largest diameter being 10 cm (Figure 1).

With respect to cardiology and cardiac surgery consult, the patient underwent aortic CT angiography. He was then admitted to the coronary care unit (CCU) for further evaluation with transesophageal echocardiogram (TEE) and transthoracic echocardiogram (TTE). Electroencephalography (EEG) and transcranial color-coded sonography (TCCS) were done. Electroencephalography (EEG) was performed during wakefulness. The posterior background consisted of normal alpha rhythm (8 Hz), with normal amplitude, reactive to eye opening and closing. Intermittent photic stimulation (IPS) and hyperventilation (HV) were performed with no abnormal discharges. No epileptic activity was observed. The study was interpreted as a normal awake EEG, although a normal EEG does not exclude epilepsy.

TCCS showed stenosis in the right and left internal carotid artery (ICA). As he had intermittent episodes of melena and hematochezia during CCU admission, he underwent endoscopy. Upper gastrointestinal (GI) endoscopy revealed a normal esophagus. The stomach showed multiple erosions in the antrum, while the cardia, fundus, and body were normal. In the duodenum, a clean-base ulcer with flat pigmented spots was observed in the bulb, while the second part of the duodenum appeared normal. A cold biopsy was taken from the antrum for histology. The final diagnosis was normal esophagus, erosive gastropathy, and duodenal ulcer. Epinephrine injection was administered. High-dose oral proton pump inhibitor (PPI) was started after the procedure.

**Figure 2:**
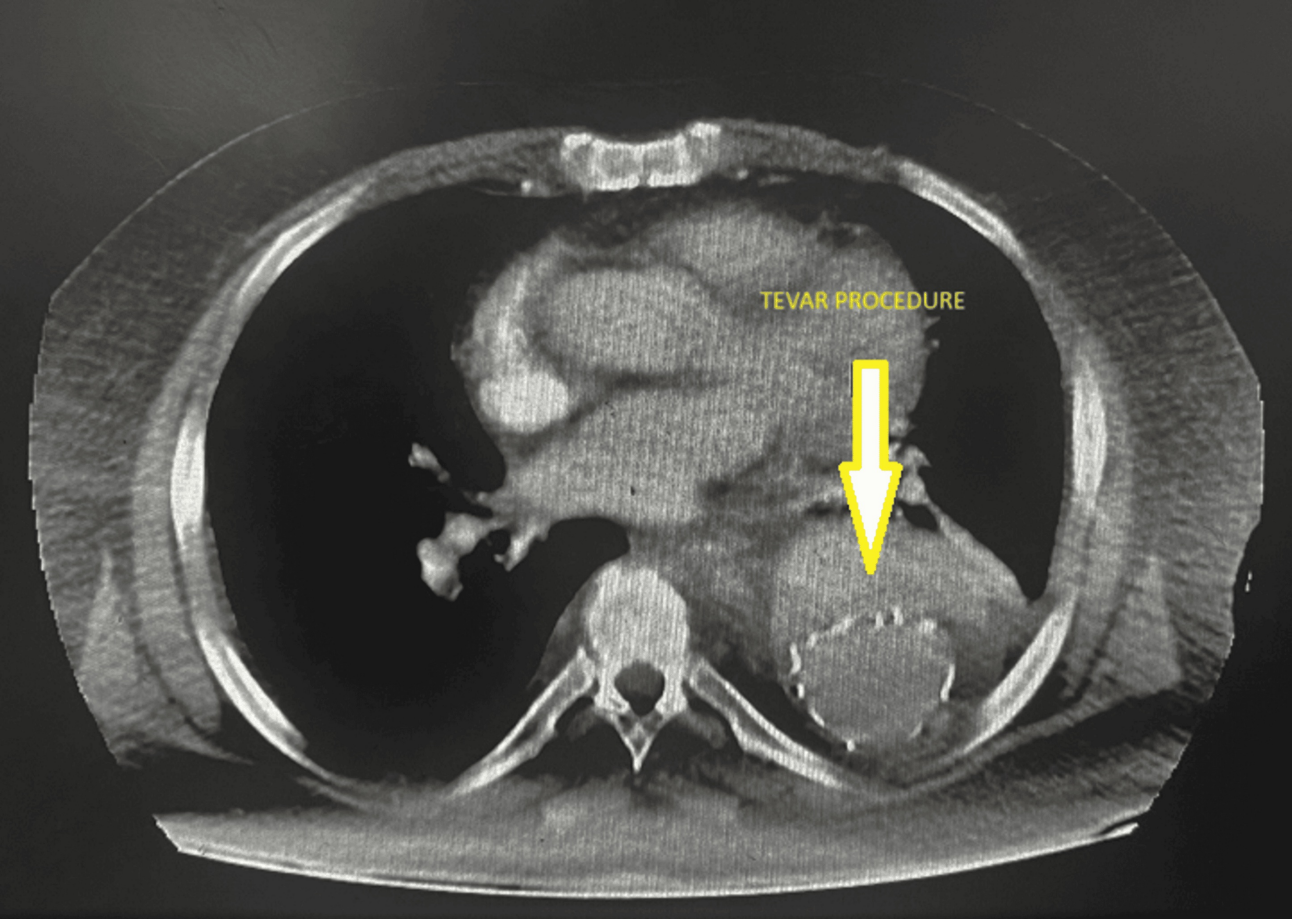
Post-TEVAR This image shows a CT scan of the thorax (axial view). The yellow dart points to a region labeled TEVAR, which indicates thoracic endovascular aortic repair. CT: computed tomography, TEVAR: thoracic endovascular aortic repair

**Figure 3:**
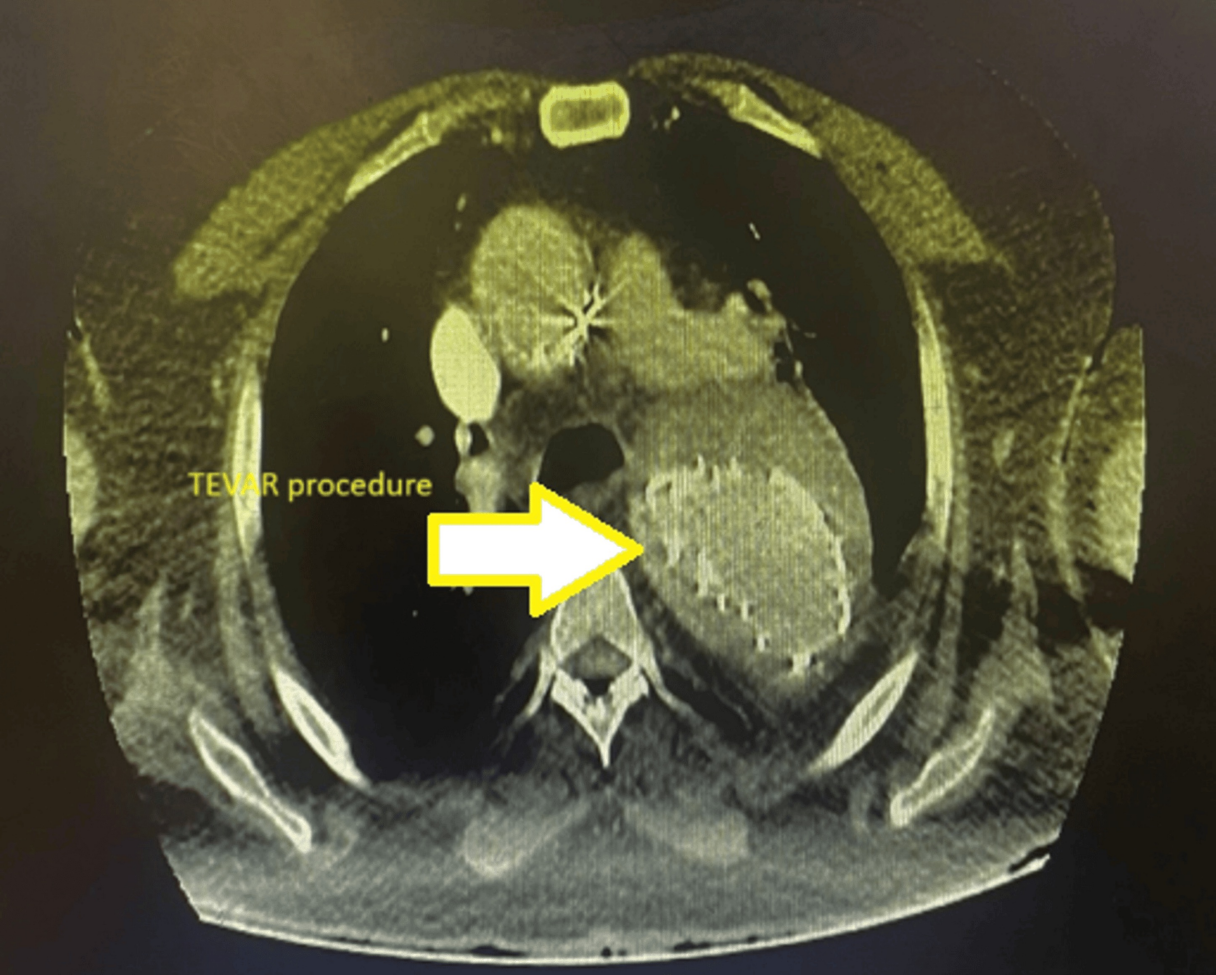
Post-TEVAR The image shows post-procedure findings. TEVAR: thoracic endovascular aortic repair

The patient underwent thoracic endovascular aortic repair (TEVAR) surgery without endoleak. The procedure was done successfully (Figure 2 and Figure 3).

He was discharged home with metoprolol 50 mg BID, acetylsalicylic acid (ASA) 80 mg daily, atorvastatin 40 mg daily, levetiracetam 500 mg daily, and phenytoin 100 mg TDS. These medications were prescribed until the next scheduled follow-up in 6-8 weeks for re-evaluation. As far as we are informed, he has not had any seizures after the surgery.

## Discussion

Aortic dissection occurs when an intimal tear causes high pressure of blood flow between the layers of the aorta, creating a true and a false lumen. Aortic dissection, as categorized by etiology, can be sporadic, genetically related, or traumatic. Depending on the duration of the dissection, there are three types described: acute, subacute, and chronic (<14 days, 15-90 days, and >90 days from symptoms onset, respectively) [2].

Aortic dissection can cause malperfusion of any organ system and thus lead to various signs and symptoms. In nearly 30% of cases, patients present with neurological symptoms, mainly stroke and syncope [3].

Aortic dissection is most commonly classified using the Stanford system. In this classification, type A involves the ascending aorta, whereas type B does not. Type A is associated with higher mortality and usually requires surgical intervention, while type B is typically managed medically [4].

Neurological complications of aortic dissection include deficits resembling ischemic stroke or coma, transient ischemic attack (TIA), spinal cord ischemia, ischemic neuropathy, and hypoxic-ischemic encephalopathy. These manifestations result from pathophysiological mechanisms such as extension of the dissection flap into the carotid or vertebral arteries, thromboembolism from the false lumen, systemic hypoperfusion due to compromised cardiac output, and hypoxic injury from reduced cerebral blood flow. Other complications include syncope, generalized tonic-clonic seizures, somnolence, and altered mental status [4].

Aortic dissection should be a consideration in patients who present with syncope, seizure, or altered mental status. In evaluating these patients, an initial chest CT scan is very important, since, according to the type of dissection and clinical presentation, immediate surgical intervention is crucial [4].

Most patients with chronic aortic dissection report episodes of TIA or transient neurological deficits. Rapid improvement in such cases is probably the result of transient arterial occlusion [5].

Conventionally, most patients with Stanford type B acute aortic dissection are treated using conservative medical treatment during the acute phase. However, in patients with complicated type B aortic dissection who present with life-threatening complications, TEVAR has been introduced as a novel and less-invasive alternative and has shown better early results than those observed with conventional therapy [6]. Recently, TEVAR was reported to be effective in not only promoting thrombosis of the false lumen but also in preventing aortic enlargement observed at long-term follow-up. TEVAR has been established as first-line therapy for complicated type B aortic dissection [7].

## Conclusions

This case highlights the importance of considering aortic dissection and aneurysm in patients presenting with atypical neurological symptoms such as recurrent seizures. In our patient, the unusual seizure presentation led to further evaluation, which uncovered a chronic type B aortic dissection with a large aneurysmal dilatation. The choice of thoracic endovascular aortic repair (TEVAR) was guided by the extent of the disease and the high risk of rupture associated with the 10 cm aneurysm. The patient's uneventful recovery and discharge without complications emphasize the effectiveness of TEVAR as a minimally invasive and beneficial treatment option in carefully selected cases.
